# Supplementary material for: Enhancing efficiency of protein language models with minimal wet-lab data through few-shot learning
Source: Nat Commun. 2024 Jul 2;15:5566. doi: 10.1038/s41467-024-49798-6 (PMC11219809; doi:10.1038/s41467-024-49798-6)
Supplement: Supplementary file 1 — Supplementary Information [file 41467_2024_49798_MOESM1_ESM.pdf]

Supplementary Information

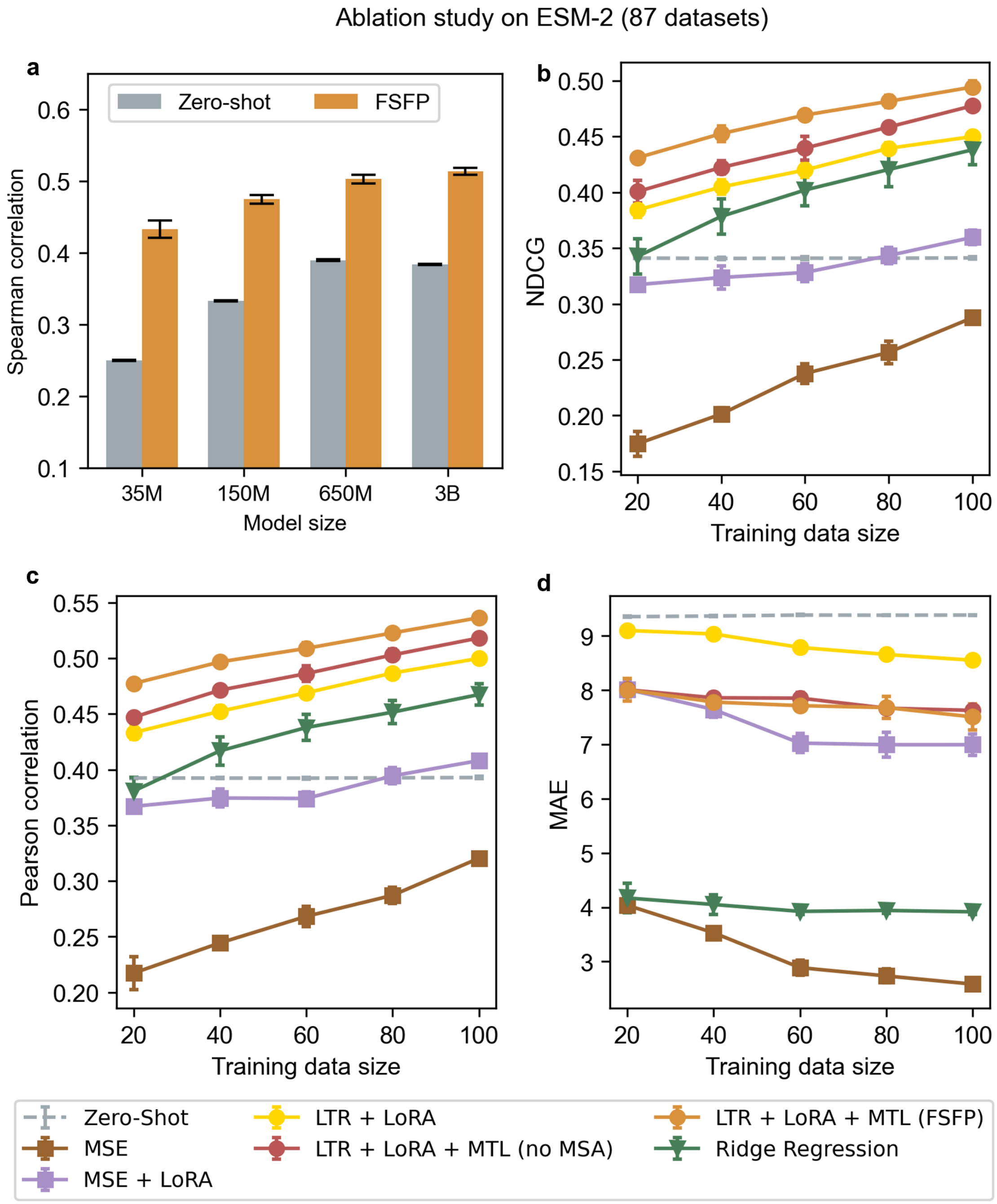

Supplementary Figure 1. Ablation study on ESM-2. a) Effect of changing the model size, with the training set size of 40. The performance is averaged across all datasets in ProteinGym, and the error bars represent the standard deviation caused by 5 random splits. The 650M model is chosen for other experiments. Average performance of different strategies is evaluated by **b)** NDCG, **c)** Pearson correlation, and **d)** MAE. Error bars represent the standard deviation caused by 5 random splits. When calculating MAE, the labels in the test set are standardized by removing the mean and scaling to unit variance. Source data are provided as a Source Data file.

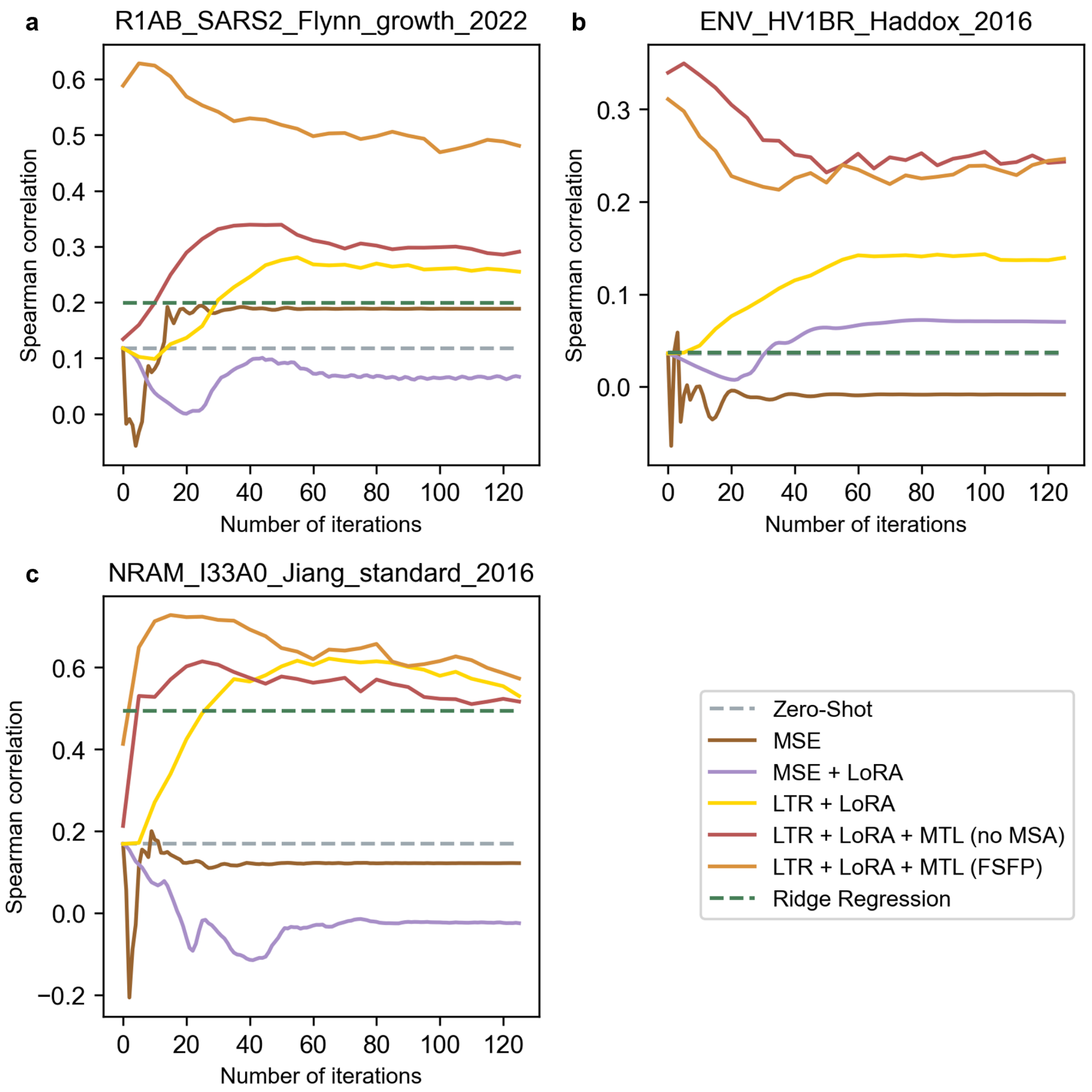

Supplementary Figure 2. Learning curves of different strategies. The performance curves of different strategies recorded on the test set of **a)** SARS-CoV-2 main protease, **b)** envelope protein Env from HIV and **c)** neuraminidase during training. The test scores here are only used for comparison and we do not access them for early stopping. The training data size is 40 in these examples. Source data are provided as a Source Data file.

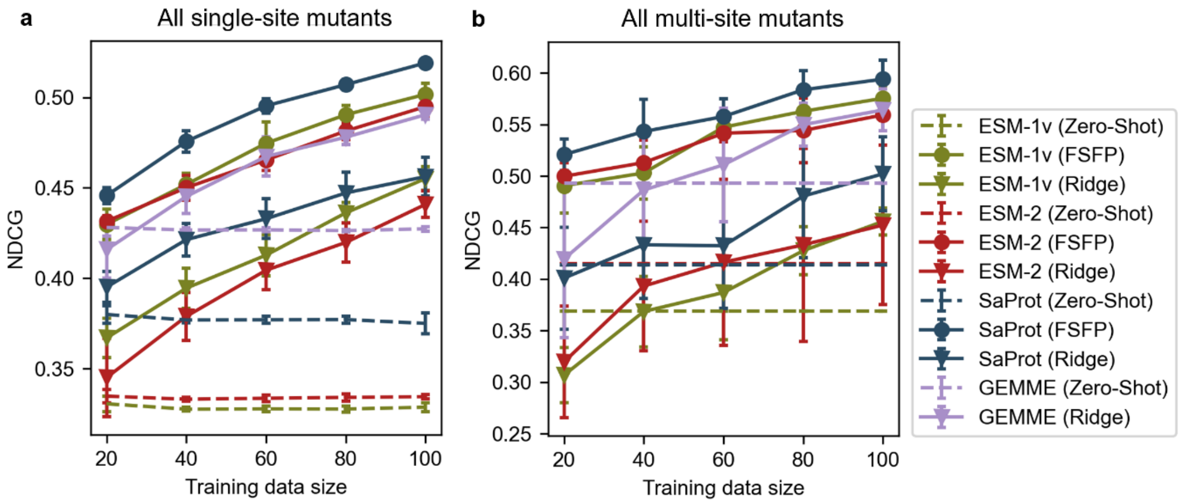

Supplementary Figure 3. Overall performance on single-site and multi-site mutants. a) Average model performance tested on single-site mutants across all 87 datasets, evaluated by NDCG. Error bars represent the standard deviation caused by 5 random splits. **b)** Average model performance tested on multi-site mutants across 11 datasets, evaluated by NDCG. Error bars represent the standard deviation caused by 5 random splits. Source data are provided as a Source Data file.

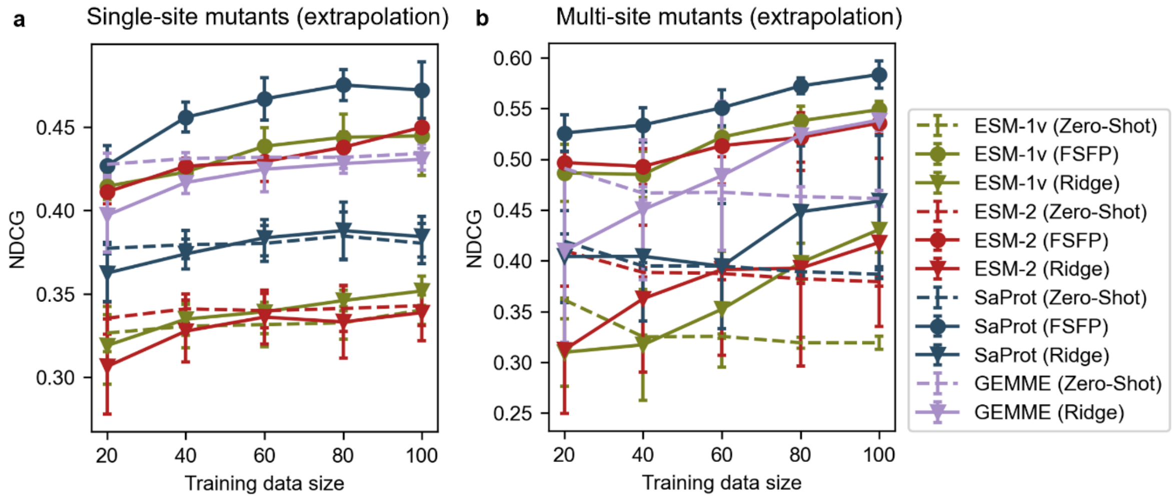

Supplementary Figure 4. Extrapolative performance on single-site and multi-site mutants. a) Extrapolating to single-site mutants whose mutated positions are not occurred in the training set, evaluated by NDCG. Error bars are centered at average performance and indicate the standard deviation caused by 5 random splits. **b)** Extrapolating to multi-site mutants whose individual mutations have no overlap with the mutations in the training data, evaluated by NDCG. Error bars are centered at average performance and indicate the standard deviation caused by 5 random splits. Source data are provided as a Source Data file.

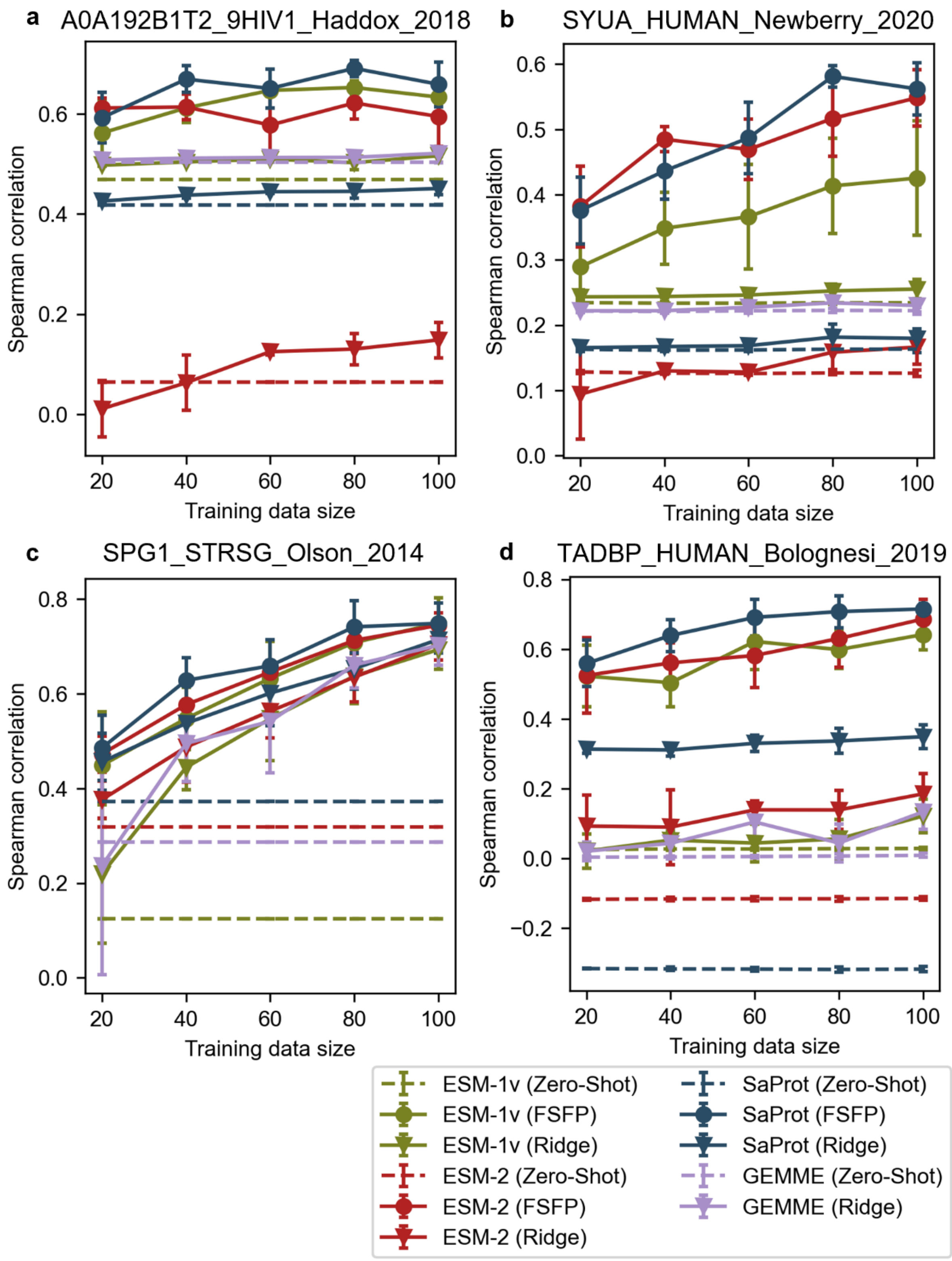

Supplementary Figure 5. Comparison of different approaches on four proteins (Spearman correlation). a) The envelope protein Env from HIV. **b)** The human α-synuclein. **c)** Protein G (GB1). **d)** The human TDP-43. The models are trained on single-site mutants and tested on all remaining data using Spearman correlation. Error bars are centered at average performance and indicate the standard deviation caused by 5 random splits. Source data are provided as a Source Data file.

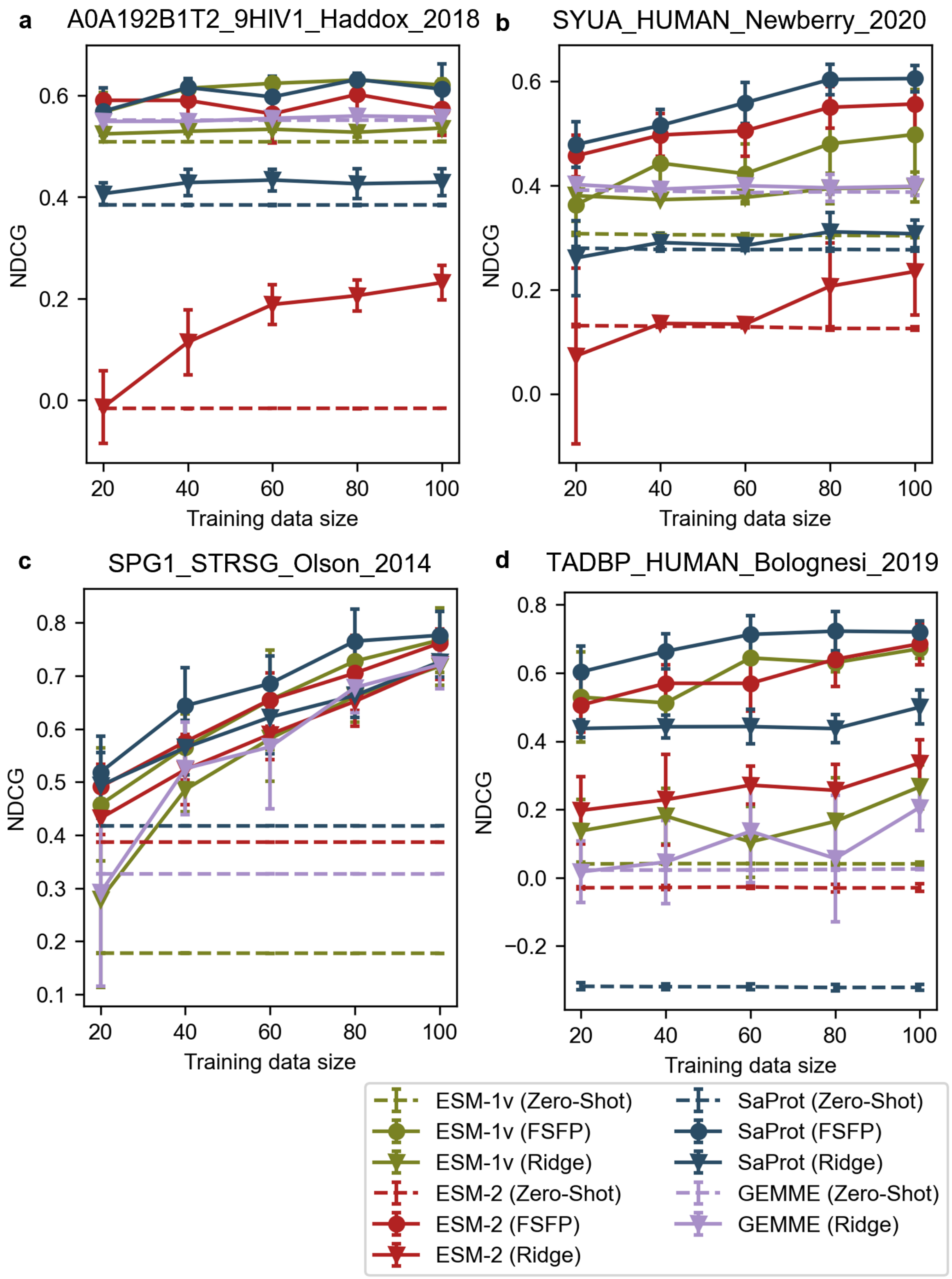

Supplementary Figure 6. Comparison of different approaches on four proteins (NDCG). a) The envelope protein Env from HIV. **b)** The human α-synuclein. **c)** Protein G (GB1). **d)** The human TDP-43. The models are trained on single-site mutants and tested on all remaining data using NDCG. Error bars are centered at average performance and indicate the standard deviation caused by 5 random splits. Source data are provided as a Source Data file.

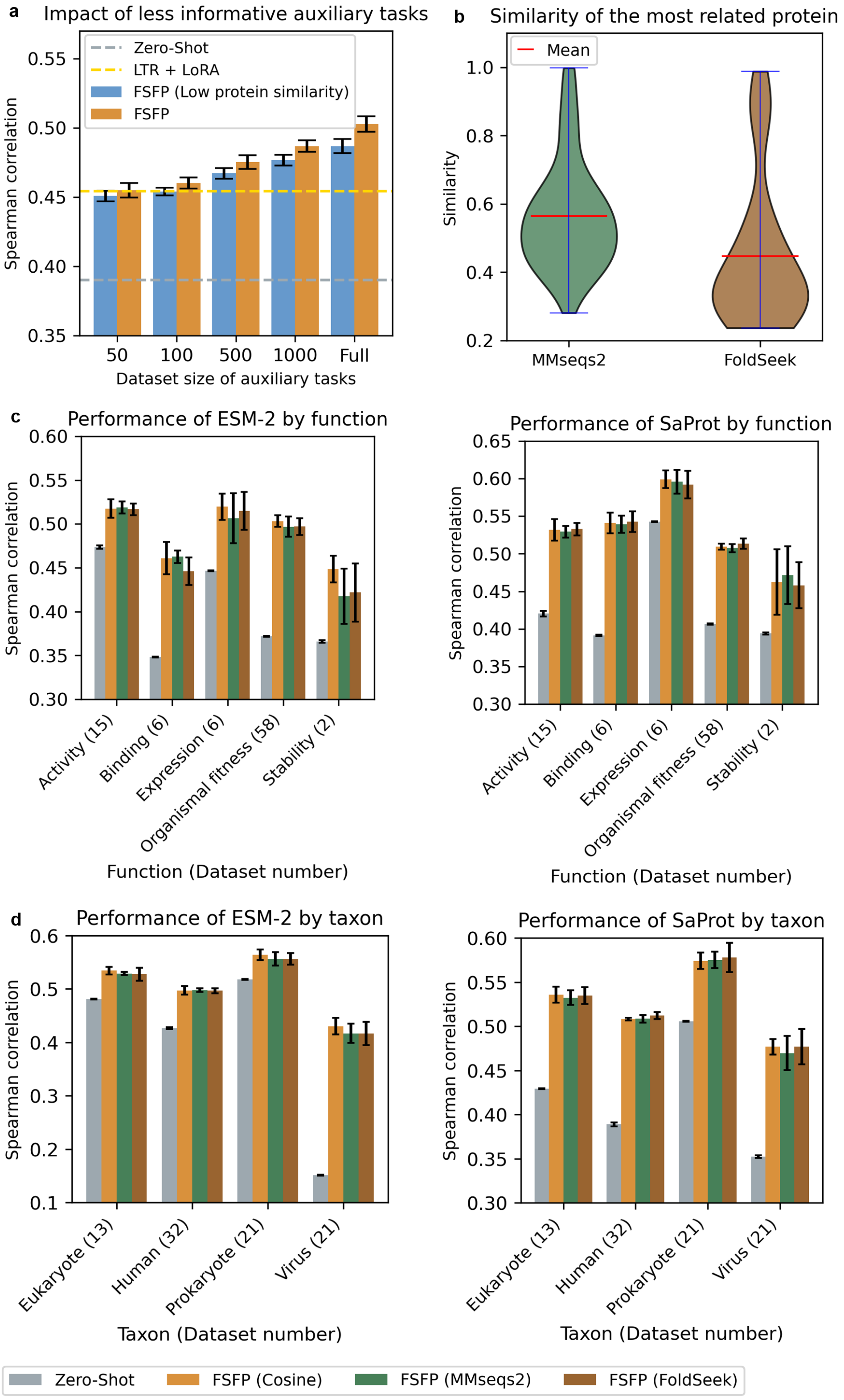

Supplementary Figure 7. Comparison of different auxiliary task selection strategies. a) Average performance of FSFP when limiting the number of mutants in the auxiliary tasks and (or) taking the labeled data from dissimilar proteins (i.e., with the lowest similarities to the target protein). The base model is ESM-2 and the target training set size is 40. Error bars represent the standard deviation caused by 5 random splits. **b)** Similarity of the most relevant protein retrieved for building auxiliary tasks, using MMseqs2 and FoldSeek respectively. **c)** Breakdown performance by the function to predict, using different methods to search similar proteins. The target training set size is 40. Error bars are centered at average performance and indicate the standard deviation caused by 5 random splits. **d)** Similar to Supplementary Figure 7c, but performance is by the taxon of the target protein. Source data are provided as a Source Data file.

Supplementary Table 1. Wet-lab experimental T_m for the single-site mutants of Phi29.

**ESM-1v (Zero-Shot)**	**T_m**	**ESM-1v (FSFP)**	**T_m**
T441L	54.38	T441L	54.38
S10I	53.75	Q55D	53.94
G245V	53.46	S551L	53.90
Q257I	53.37	V19P	53.86
V130L	53.09	L567E	53.61
P129S	52.82	G245V	53.46
V54N	52.67	V566E	53.38
Wild type	***52.64***	V130L	53.09
C290K	52.58	S551M	53.04
Q257V	51.97	H3K	52.92
Q257A	51.97	F526L	52.84
W367R	51.74	T140P	52.76
Q257L	51.27	***Wild type***	***52.64***
Y449G	51.11	C290K	52.58
V54E	51.04	P558W	52.56
M30Y	51.03	V566K	52.50
Y369E	50.51	V568K	52.40
W327D	49.90	Y224D	52.23
C530K	49.30	P404E	52.20
H35G	48.97	M506T	51.76
W327K	48.51	T542Y	51.21

The mutants are the top 20 predictions from ESM-1v before and after trained by FSFP respectively.

Supplementary Table 2. Performance of ESM-2 (FSFP) under different MAML settings.

MAML setting		Spearman correlation
$\alpha = 0.005$	$g = 2$	0.503 ± 0.004
	$g = 3$	0.503 ± 0.001
	$g = 4$	0.500 ± 0.003
	$g = 5$	0.503 ± 0.006
	$g = 6$	0.500 ± 0.006
$g = 5$	$\alpha = 0.1$	0.487 ± 0.008
	$\alpha = 0.05$	0.490 ± 0.007
	$\alpha = 0.01$	0.496 ± 0.004
	$\alpha = 0.005$	0.503 ± 0.006
	$\alpha = 0.001$	0.503 ± 0.004

Average performance across all datasets in the benchmark are reported, along with the standard deviation caused by 5 random splits. The training set size is 40. α and g is the gradient step size and number during the inner loop of MAML.

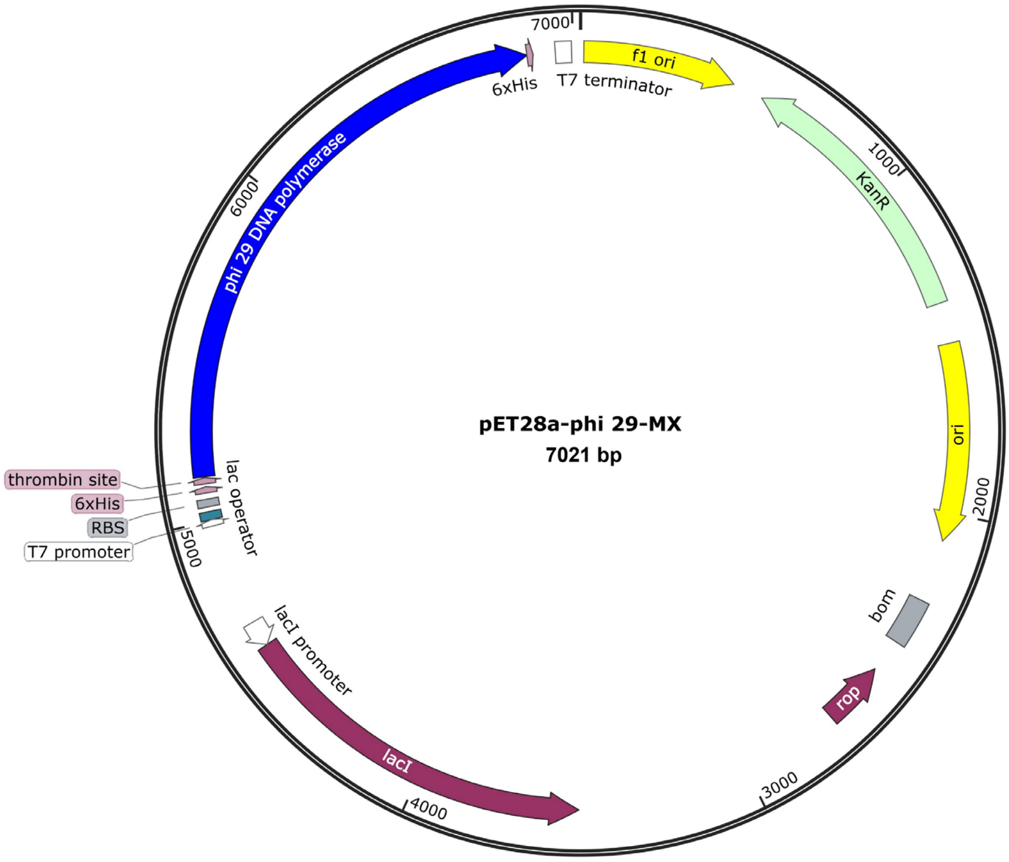

Supplementary Figure 8. Schematic diagram of plasmid pET28a-phi 29-MX.
