# Supplementary material for: Enhancing efficiency of protein language models with minimal wet-lab data through few-shot learning
Source: Nat Commun. 2024 Jul 2;15:5566. doi: 10.1038/s41467-024-49798-6 (PMC11219809; doi:10.1038/s41467-024-49798-6)
Supplement: Supplementary file 3 — Description of Additional Supplementary Files [file 41467_2024_49798_MOESM3_ESM.docx]

**Description of Additional Supplementary Files:**

**Supplementary Data 1**: Detailed performance of different approaches on each dataset measured by Spearman correlation.

**Supplementary Data 2:** Detailed performance of different approaches on each dataset measured by NDCG.
